# Visual attention span performance in German-speaking children with differential reading and spelling profiles: No evidence of group differences

**DOI:** 10.1371/journal.pone.0198903

**Published:** 2018-06-18

**Authors:** Chiara Banfi, Ferenc Kemény, Melanie Gangl, Gerd Schulte-Körne, Kristina Moll, Karin Landerl

**Affiliations:** 1 Institute of Psychology, University of Graz, Graz, Austria; 2 Department of Child and Adolescent Psychiatry, Psychosomatics and Psychotherapy, Ludwig-Maximilian University, Munich, Germany; 3 BioTechMed-Graz, Graz, Austria; Katholieke Universiteit Leuven, BELGIUM

## Abstract

An impairment in the visual attention span (VAS) has been suggested to hamper reading performance of individuals with dyslexia. It is not clear, however, if the very nature of the deficit is visual or verbal and, importantly, if it affects spelling skills as well. The current study investigated VAS by means of forced choice tasks with letters and symbols in a sample of third and fourth graders with age-adequate reading and spelling skills (*n* = 43), a typical dyslexia profile with combined reading and spelling deficits (*n* = 26) and isolated spelling deficits (*n* = 32). The task was devised to contain low phonological short-term memory load and to overcome the limitations of oral reports. Notably, eye-movements were monitored to control that children fixated the center of the display when stimuli were presented. Results yielded no main effect of group as well as no group-related interactions, thus showing that children with dyslexia and isolated spelling deficits did not manifest a VAS deficit for letters or symbols once certain methodological aspects were controlled for. The present results could not replicate previous evidence for the involvement of VAS in reading and dyslexia.

## Introduction

Letter strings can be read with two different visual processing modes according to the multi-trace model of polysyllabic word reading [1]. The global mode is supposed to rely on a broad visual-attention window, which allows the simultaneous analysis of the string in parallel, with low phonological short-term memory load. The analytic mode represents instead a serial procedure with a high phonological short-term memory load, where the visual-attention window is narrowed down to only part of the string and is sequentially shifted from left to right. In this theoretical framework, Valdois, Bosse and Tainturier [2]—hypothesized that phonological deficits affecting the analytic mode should primarily impair pseudoword reading. However, such a deficit in decoding would in turn affect self-teaching strategies and the build-up of word specific representations [3], resulting in a mixed dyslexia profile where both pseudoword and word reading are deficient. On the contrary, a reduction in the visual-attention window may induce difficulties in the global mode, thus impairing the build-up of a wide orthographic lexicon. This type of visual-attention deficit is supposed to cause problems at the level of lexical reading strategies for irregular words while sublexical decoding may be unimpaired [2]. However, severe reductions of the visual-attention window might also negatively impact on regular word and pseudoword reading, resulting in a mixed dyslexia profile [2,4]

The visual attention span (VAS) is usually investigated by means of two types of oral report tasks presenting a five-element string for a short time window. In whole-report paradigms, participants are asked to name as many elements as possible from the previously viewed string. In partial-report paradigms, the task is to report verbally a single element of the string that occurred at a cued position. Several studies on such “classical” paradigms showed generally poorer performance in dyslexic individuals compared to typically developing children (TD) and were thus taken as evidence in favor of a VAS deficit in dyslexia (see S1 Appendix for details). Additionally, performance on the VAS task was shown to be related to reading measures for regular and irregular words and pseudowords, and regression models showed that VAS and phonological awareness (PA) represent two independent predictors of reading measures [5–7]. It was thus claimed [5] that VAS and phonological impairments dissociate within dyslexia samples.

It is not clear, however, if a VAS deficit should be expected for poor readers only or if it relates to spelling as well. The multi-trace model [1] relates VAS deficits specifically to reading development and failure, and deficits in spelling can perhaps be interpreted as a consequence of the reading failure [4]. However, a considerable percentage of children have marked deficits in spelling in spite of age-adequate reading skills, especially in orthographies with asymmetric grapheme-phoneme and phoneme-grapheme correspondences [8]. German orthography, for instance, is highly consistent in the reading direction, whereas phoneme-grapheme correspondences are often inconsistent, so that the speller must retrieve the correct letter sequence for a certain word from long-term memory. Children with isolated spelling deficits seem to have intact decoding skills, as they are reading words and pseudowords accurately and fluently and their spelling errors typically present phonologically adequate translations of the sound sequence [9,10]. Still, they do not have orthographic representations at their disposal, which would allow them to produce the correct spelling. The question thus arises as to why individuals with an isolated spelling deficit experience difficulties in developing a large orthographic lexicon, although the input in terms of reading is intact. From the VAS perspective, it is possible that the build-up of orthographic representation in poor spellers is caused by a deficient global mode due to a reduced visual attention window. In order to address this issue, we expanded the standard group design of comparing dyslexic and typically developing children with an age-matched group of children with isolated spelling disorder.

Two methodological issues have been controversially discussed with respect to tasks developed to assess VAS. First, such tasks often rely on verbal stimulus material (letters or digits), and it was argued [11], that group differences reported on verbal (but not on nonverbal) stimulus material, might be explained by other already established cognitive deficits associated with dyslexia, such as the visual-to-verbal mapping impairment assessed by rapid automatized naming (RAN) tasks. Such a link was not always confirmed [12]. Others, however, reported impaired performance among dyslexic participants for verbal as well as non-verbal material [13] and therefore claimed that the very nature of the VAS deficit is visual, not verbal. In light of this controversy, we chose to include a verbal as well as a nonverbal condition in the current study.

Second, the choice of oral reports might constitute an additional disadvantage for poor readers, given their established impairment in verbal response latencies [14]. Additionally, individuals with dyslexia often show reduced phonological short-term memory [15], which might inflate poor performances on oral report tasks on verbal material. Studies using non-verbal stimuli like symbols or pseudoletters and relying on other paradigms than oral reports (e.g., two-alternative forced choice tasks– 2AFC), often failed to find differences in the performance of poor readers compared to typical readers [11,12,16,17], with at least two exceptions where poorer performance in dyslexia was reported [18,19]. However, those studies used very different experimental paradigms, with array recognition vs. character recognition tasks, low vs. high discriminability stimuli, short vs. long presentation times for the array (for a more detailed overview of the existing literature, see S2 Appendix). Such setups place different loads on visuo-spatial attention and short-term memory, which could explain inconsistent results. However, while Yeari, Isser and Schiff [20] recently showed differential task effects, these effects were similar for typical and poor readers. Yeari et al.[20] pointed out that the only two studies that reported deficient performance in dyslexia samples [18,19] did not explicitly exclude individuals with comorbid ADHD. Obviously, broader attentional deficits as associated with ADHD would negatively impact on performance in any VAS task.

An alternative hypothesis holds that the VAS deficit is due to enhanced crowding associated with reading difficulties [12]. Note, however, that a VAS impairment was observed in dyslexia even when the impact of crowding was minimized [21]. Crowding refers to the negative effect of surrounding visual elements on the recognition of the target [22]. It affects poor readers’ speed more than TD children’s, since they require a larger critical spacing to reach a maximum reading speed [23,24]. Collis et al. [12] suggested that group differences should be expected on the most “crowded” positions of a five-element string, i.e. the second and the fourth, if the poor performance on the VAS span task was explained by excessive crowding in dyslexia. However, this expectation would be valid only if we assume that participants are fixating the middle of the string during its brief presentation. Interestingly, none of the previous studies included a method to control for eye fixations. In some cases, better performance for the leftmost position is reported in western left-to-right orthographies such as German [16] and English [12], whereas lower performance on the left than in the center was reported on Hebrew speaking participants [20]. However, without a systematic control of eye-movements, it might be possible that participants were already focusing on the left when the string appeared, thus explaining the higher performance found for the leftward element.

An important advantage of the present study is the reliance on the eye-tracking technique as a mean to control participants’ eye-movements. To run the experiment, participants had to start each trial fixating the center of the five-element string, i.e. the element on the third position. This way, we could ensure that their eyes were not already shifted to the left. Besides, we used a forced choice paradigm to avoid the high phonological load of oral reports.

To summarize, the present study tested the VAS deficit account of dyslexia. We investigated if a VAS deficit is present in German-speaking children with reading and/or spelling disorders on letters as well as symbols. First, we tested if the VAS deficit is specifically related to reading or if it affects also children with impaired spelling and age-appropriate reading skills as could be predicted by the VAS deficit account. Consistently, we expected the VAS performance to be independent of verbal and nonverbal IQ, working memory and phonological awareness. Excessive crowding might provide an alternative explanation for poor performance on the letter and symbol tasks. We thus reasoned that the presence of impaired performance exclusively on the most „crowded”positions 2 and 4 would hint for the presence of excessive crowding in the deficits groups.

In contrast to the VAS deficit account, the visuo-verbal access deficit account of dyslexia predicts that children with reading and/or spelling problems show impairments in VAS letters only, while performance in VAS symbols should be unimpaired. Furthermore, performance on VAS letters (but not VAS symbols) should correlate with RAN.

## Materials and methods

### Participants

The study was approved by the ethics committee of the University of Graz and by the institutional review board of Medical Faculty of the University Hospital Munich. It was performed in accordance with the latest version of the Declaration of Helsinki and in compliance with national legislation. Written informed consent was obtained on behalf of the children from their parents. Children were selected based on an extensive classroom screening with 4123 children at the end of 3^rd^ and beginning of 4^th^ Grade.

Data collection was carried out in two collaborating sites: Munich (Germany) and Graz (Austria). Standardized classroom tests of sentence reading fluency (SLS 2–9: [25]) and spelling (DRT 3: [26]) were used. Children were also individually administered a standardized one-minute word and pseudoword reading speed test (SLRT-II: [27]), either in school or in the lab. From this large sample, we selected children with dyslexia if they had percentile ≤ 20 on two reading measures and on the spelling measure. Note that one child in the dyslexia group had not participated in the classroom screening but received the SLRT-II and an age adequate standardized spelling test (DRT 4: [28]) during an individual assessment. We also included three children in the dyslexia group whose reading performance was ≤ percentile 20 on all three reading tests while their spelling level was somewhat above percentile 20, but at or below percentile 25 in all three cases. Children were selected for the group of isolated spelling disorder if they showed spelling performance ≤ percentile 20 and age-adequate reading, i.e. percentile ≥ 25 on the mean of the three reading measures (sentence, word and pseudoword reading). Children with typical reading and spelling skills had percentiles between 25 and 85 on the mean of the three reading measures and on spelling.

All children had German as their first language, a non-verbal IQ ≥ 85 (CFT 20-R: [29]), normal or corrected-to-normal vision, no identified sensory or neurological deficits, no clinical ADHD diagnosis as well as an above-threshold score on a parental questionnaire for attention deficits (DISYPS-II: [30]).

Altogether 177 children were assessed: 72 typically developing children (TD), 56 children with dyslexia and 49 children with isolated spelling deficit (SD). Due to a programming error, which is described in detail below, 76 participants received non-randomized versions of the VAS-task, and were excluded from the main analyses. The final dataset comprised 101 participants: 43 typically developing children (TD), 26 children with dyslexia and 32 children with isolated spelling deficit (SD).

### Literacy and cognitive measures

The tasks described in this paper were part of a larger task battery comprising an initial screening in school, including the classroom measures of sentence reading and spelling. A nonverbal IQ test was administered either individually or in the group. The individually administered word and pseudoword reading test was conducted in school or in the lab. All other tasks were carried out as part of three to four individual assessments in our labs, each of which lasted between 90 and 120 minutes. The tasks described here were usually carried out during the first and second assessments, which took place between 2 to 12 weeks after the classroom screening.

#### Reading

In the classroom-administered standardized reading speed test (SLS 2–9: [25], parallel test reliability is .95 for Grade 2 and .87 for Grade 8), children were asked to silently read single-line-long sentences with simple semantic and syntactic structure (e.g., “Trees can speak”). They had to mark each sentence as right or wrong by circling a check mark or a cross at the end of the line. The task was terminated after three minutes. The raw score was the number of correctly marked sentences.

In the individually administered one-minute reading speed test (SLRT-II: [27], parallel test reliability is .94 for words and .90 for pseudowords), children were instructed to read aloud a word and a pseudoword list as fast as possible without making errors. The raw score consisted in the number of correctly read items in one minute.

#### Spelling

The standardized classroom spelling test (DRT 3: [26]; split-half reliability is .95) comprised 44 words that had to be written into sentence frames. The experimenter dictated each word, then read out the full sentence and then repeated the word again. The number of correct word spellings was scored.

#### Nonverbal IQ

The first part of the German version of the Culture Fair Intelligence Test (CFT 20-R: [29]; test reliability = .92 according to manual) was given as an estimate of nonverbal IQ. Its four subtests comprised Series, Classification, Matrices and Topology.

**Vocabulary** was assessed by the Vocabulary subtest of the German version of the Wechsler Intelligence Scale for Children (WISC-IV: [31]).

**Verbal short-term and working memory** were investigated by the Digit Span subtest of the German version of the Wechsler Intelligence Scale for Children (WISC-IV: [31]).

**Processing Speed** was investigated by the Symbol Search subtest of the German version of the Wechsler Intelligence Scale for Children (WISC-IV: [31]).

**Phonological Awareness (PA)** was assessed by means of a computerized phoneme deletion task running on Presentation 16.3 (Neurobehavioral Systems, Inc., Berkeley, CA, USA). The task consisted of four practice trials and 25 test trials (20 mono- and 5 disyllabic pseudowords) which were presented via headphones. Children were asked to repeat each pseudoword first and then to pronounce it without a specified phoneme (e.g., “/folt/ without /t/”). Any pseudoword that children could not pronounce correctly was played again up to two times. Items that were still not repeated correctly were excluded from analysis (about 9% of the items). The ratio of correct responses to the total number of responses was taken into account. Cronbach’s alpha was .76.

#### Rapid automatized naming (RAN)

Standard paradigms of RAN-objects and RAN-digits [32] were presented. Both conditions required to name a matrix of 40 items as quickly and accurately as possible. Simple pictured objects and digits were presented on separate sheets in five columns and eight lines. Item order was randomized and each item was presented once in each line. Children were familiarized with each condition with a 3 x 5 RAN array format. The time needed to name the full item set and any occurring errors were recorded and transformed into items named correctly per second. The correlation between conditions was .46, which corresponds to earlier studies [33].

#### ADHD-rating

Parents were asked to answer a standardized questionnaire (DISYPS-II: [30]) which consists of 20 items with a 4-point rating scale investigating symptoms of inattention (9 items), hyperactivity (7 items) and impulsivity (4 items). A high score on the questionnaire is indicative of high ADHD symptoms. Cut-off score for males is 1.60, for females 1.15.

### VAS Tasks

#### Apparatus

The VAS task was run inside a dimly lit room and was controlled with Experiment Builder software (RS Research, version 1.10.1241). Children were seated in front of a computer screen (Graz: 120-Hz refresh rate, 1024 x 768 pixels; Munich: 120-Hz refresh rate, 1280 x 960 pixels) at a viewing distance of about 65 cm. To ensure that children looked at the central fixation cross, eye movements were monitored using an EyeLink 1000 tower mount eye tracker in Graz and an EyeLink 1000 Plus desktop mount eye tracker in Munich (SR Research, Toronto, Canada). Eye-movement data were not further analyzed.

#### Stimuli

**Fig 1:**
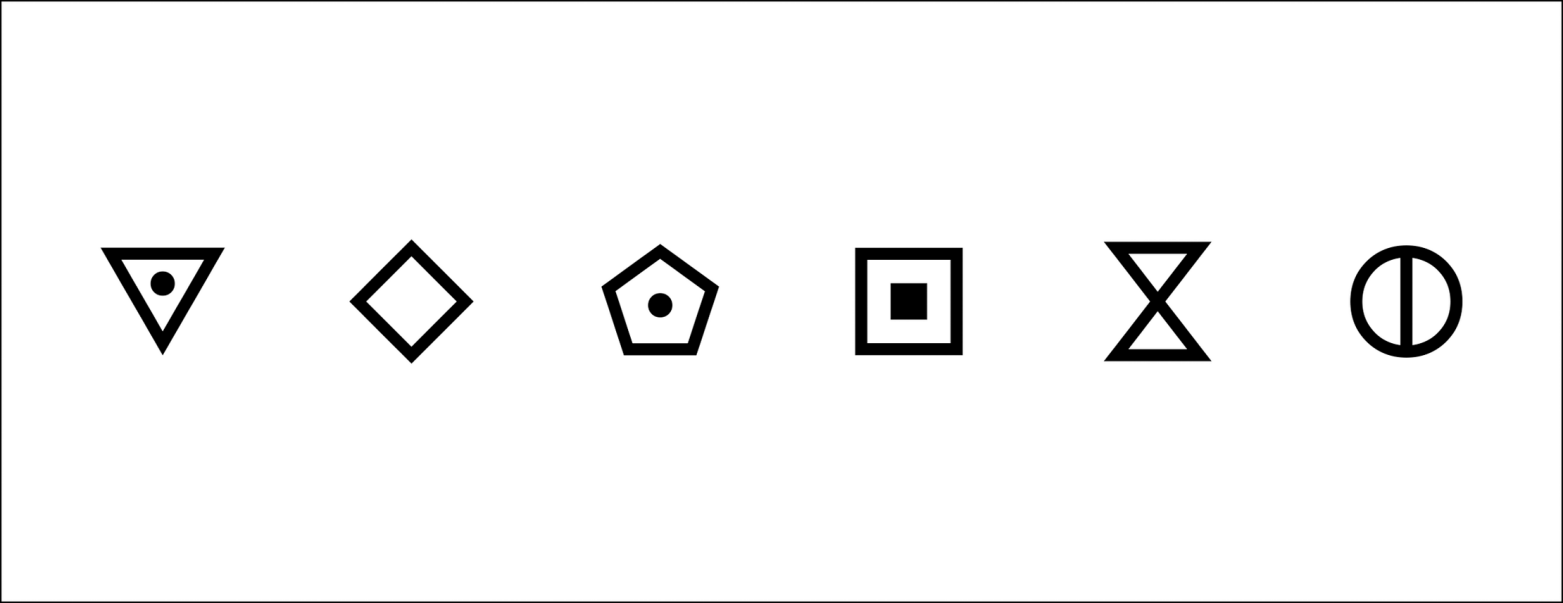
Stimuli used in the symbol task.

Stimuli were black on a white background and consisted of five-element arrays of letters or symbols with five position lines beneath them. They subtended 3° of visual angle. In the letter task, five out of six uppercase letters (D, F, H, M, R, S) were used with Arial Monospaced font. They had the same height and width. To avoid excessive crowding, the inter-letter distance was set to 11% as in Lobier, Zoubrinetzky and Valdois [13], which corresponds to a visual angle distance of 0.32°. The design of the symbols task was identical with the use of six different symbols from the SPSS Marker Set font, which had the same height and width. Symbols were slightly bigger than letters, thus yielding inter-element spacing of 0.28° of visual angle. However, note that the five-element strings for letters and symbols had the same overall width. Fig 1 depicts the six symbols used.

Each task (letters, symbols) comprised 60 trials. A trial constituted of a five-element array immediately followed by a single-element target. The child’s task was to say “yes” if the target appeared on the same position as in the array. If the target was not included in the array, the correct oral response was “no”. Yes and no responses were balanced across trials. Each of the six letters or symbols appeared once as a target on each position. This allowed us to analyze performance for the correct identification of the target on each position. Note, however, that the target was never included in the array when the expected response was no. We could thus not analyze position errors, defined as the correct identification of the target on the wrong position.

The order of the experimental trials was pseudo-randomized with the restriction that no more than two trials with the same expected response appeared in immediate succession. Four equivalent pseudo-randomized versions of the experiment were prepared and randomly assigned to the participants.

#### Procedure

**Fig 2:**
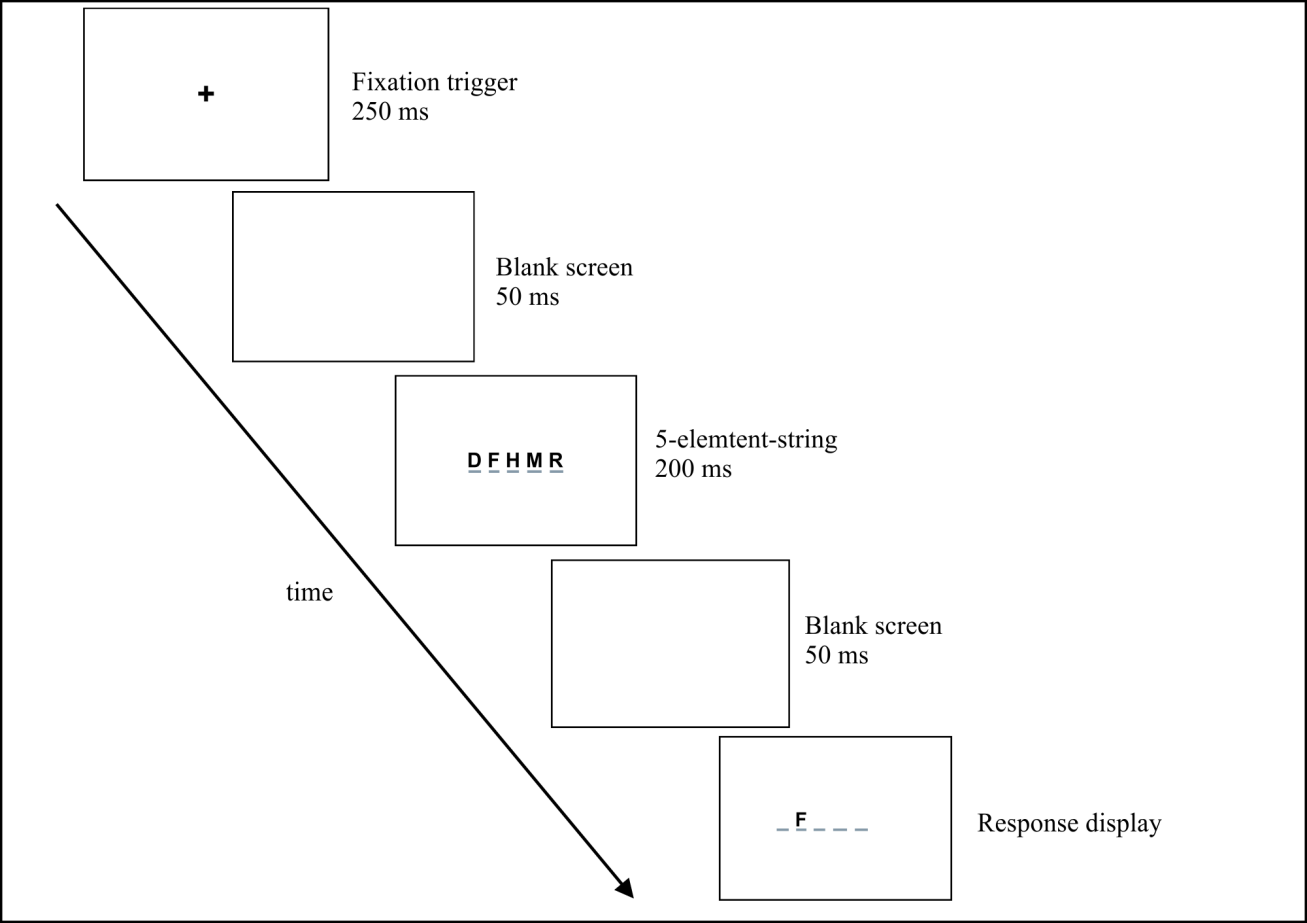
A trial from the VAS task with letters.

Fig 2 shows an example of the VAS task for letters. Children received oral instructions accompanied by visual examples as well as six practice trials. The VAS experiment started with a nine-point calibration of the eye-tracking system, which was used to ensure that participants looked at the fixation cross at the beginning of each item. Each trial began with a slide showing a central fixation cross, which was connected to a fixation trigger. The trial went further only if the participants fixated the cross for 250 ms. A blank screen appeared for 50 ms, followed by the presentation of the five-element string for 200 ms. After a 50 ms blank screen, a single-element target was shown on one of the five positions, and remained on screen until response. Children responded verbally with “yes” or “no”. Oral responses were recorded by the experimenters by mouse-clicking. After a blank screen appearing for 1000 ms, a new trial began. In case of a fixation trigger failure, children underwent a new calibration procedure and continued the experiment from the point where it was interrupted.

#### Data preprocessing

Note that data were not available on the VAS symbols condition for one child of the TD group. The present dataset contained an equal number of expected “yes” and “no” responses, thus allowing the computation of *d*-prime (*d’*) measures based on the Signal Detection Theory [34]. Hit rates (number of yes responses divided by expected yes responses) and False Alarm rates (number of falsely given yes responses divided by expected no responses) were calculated for each position for the letter as well as the symbol tasks. To allow the correct calculation of *d’* for each participant, Hit and False Alarm values of zero and one were corrected to 1/N and (N-1)/N, respectively. The *d’* is defined as *Z*(Hit Rate)—*Z*(False Alarm Rate), where *Z* is the inverse cumulative of the normal distribution. The *d’* was computed in IBM SPSS Statistics 24 with the IDF.NORMAL function, for each position for each task.

During analysis, it turned out that three of the pseudo-randomized versions of the tasks were not implemented as planned in one of the participating labs due to an experimental error. As a consequence, for 76 children (29 TD, 30 children with dyslexia, 17 SD), some letters and symbols appeared several times in the same position while others were not presented as targets. As we were seriously concerned that results might be influenced by the non-balanced presentation, we decided to apply a conservative approach and report the main analyses based on those 101 participants who received correct versions of the VAS only. For the sake of transparency, we also provide the relevant information on the full sample of 177 children.

## Results

### Literacy and cognitive measures

Table 1 summarizes the literacy variables for the reduced sample of 101 children who received the VAS tasks without experimental error. Performance on the literacy measures reflected our selection criteria: children with dyslexia showed lower performance than the TD and SD groups on sentence, word and pseudoword reading, while both deficit groups had lower spelling scores than TD children. The SD group showed age-adequate reading performance, which did not differ from TD children. Furthermore, the two deficit groups did not differ in spelling (the same analysis was run on the original sample of 177 children, including those who had received non-balanced VAS tasks. The group differences on our selection criteria were similar to the ones reported in Table 1, all *p*s < .001, *ES* between .52 and .83). Although a selection criterion of percentile 20 for poor performance was lenient, Table 1 shows that mean performance was around percentile 10 for reading and spelling measures in the deficit groups. With one exception, all 10 children in the dyslexia group who were recruited in Munich had a clinical dyslexia diagnosis according to the German diagnostic guidelines [35], i.e. percentile ≤ 16 in the individually administered word reading test (27) and IQ ≥ 70. Fourteen of 16 children in the dyslexia group who were recruited in Graz also fulfilled these criteria. However, the Austrian school system does not recognize any formal diagnosis.

**Table 1:** Mean scores (M) and Standard Deviations (SD) for literacy and cognitive measures in the three groups.

	TD *n* = 43		Dyslexia *n* = 26		SD *n* = 32					
	*M*	*SD*	*M*	*SD*	*M*	*SD*	*F*	*p*	*ES*	Post-hoc comparisons
Age (months)	112.16	3.98	113.15	7.07	114.41	6.86	1.34	.27	.03	
Sentence reading percentile (SLS)	53.40	15.03	8.76	7.84	52.03	14.54	100.82	< .001	.68	TD = SD > dyslexia
Word reading percentile (SLRT-II)	51.00	14.90	8.69	7.20	45.75	18.36	73.25	< .001	.60	TD = SD > dyslexia
Pseudoword reading percentile (SLRT-II)	54.14	16.92	12.27	8.53	48.75	22.34	51.44	< .001	.51	TD = SD > dyslexia
Spelling percentile (DRT-3)	50.60	13.24	9.81	7.24	13.13	5.01	195.24	< .001	.80	TD > dyslexia = SD
Nonverbal IQ (CFT-20)	104.67	10.42	102.19	11.88	101.06	9.96	1.13	.33	.02	
WISC-IV (standard score):										
Vocabulary	11.67	3.10	11.19	3.23	10.84	3.16	.65	.52	.01	
Digit Span	10.47	2.45	10.23	3.05	9.78	2.06	.69	.51	.01	
Symbol Search	11.65	2.11	10.58	2.08	11.28	2.51	1.87	.16	.04	
Phonological Awareness (% correct)	.85	.12	.65	.18	.76	.15	15.15	< .001	.24	TD > SD > dyslexia
RAN digits/s	2.08	.40	1.71	.36	1.99	.47	6.66	.002	.12	TD = SD > dyslexia
RAN objects/s	1.08	.17	.91	.13	1.05	.18	8.23	< .001	.14	TD = SD > dyslexia
ADHD Questionnaire Score	.39	.31	.56	.43	.71	.37	7.10	.001	.13	TD < SD

There were no significant group differences on age, nonverbal IQ, and WISC-IV Vocabulary, Digit Span and Symbol Search subtests. On phonological awareness, the dyslexia group had the lowest score. The SD group showed higher PA performance than the dyslexia group, but lower than the TD group, which yielded the highest score. On both RAN tasks, the dyslexia group had lower scores than the TD and SD groups, which did not differ from each other. The ADHD score was largely comparable between TD and dyslexia groups, while it was significantly higher (indicating more ADHD-symptoms reported by parents) in SD compared to TD children. However, note that all children had ADHD-scores within the normal range.

### VAS task

#### Group comparison on target identity

In a first step, we considered whether groups differed on accuracy for the six different letters/symbols that were used as targets. A 3 x 2 x 6 ANOVA was run on accuracy, with group (TD, dyslexia, SD) as between subjects factor, task (letters vs symbols) and stimulus identity (the 6 different letters/symbols) as within subjects factors. Importantly, neither the group main effect nor any interactions involving group were significant (all *Fs* < 1).

#### Group comparison on positions

**Fig 3:**
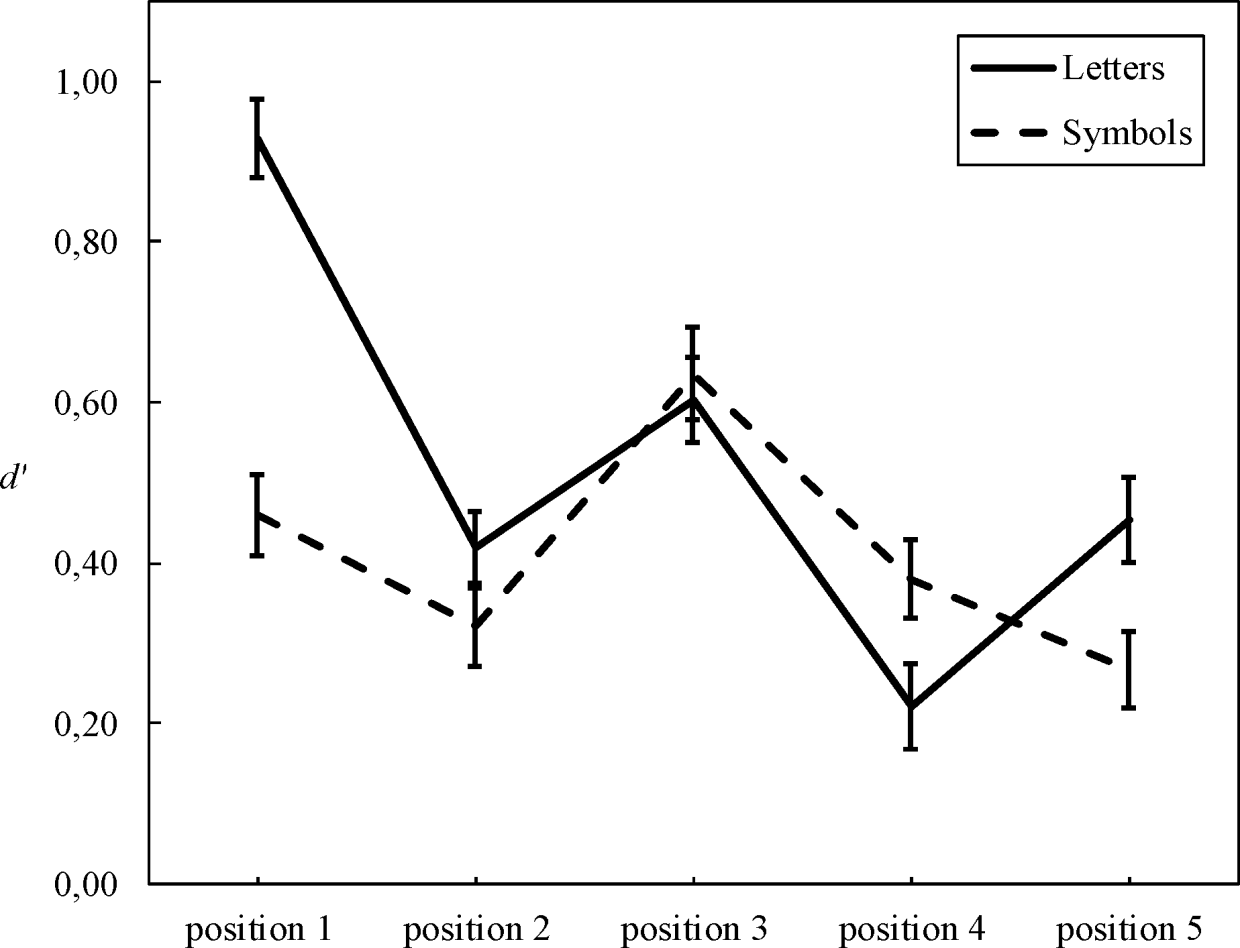
*d’* as a function of position for the letter and symbol tasks. Bars represent standard errors.

A 3 (group: TD, dyslexia, SD) x 2 (task: letters vs. symbols) x 5 (position) ANOVA was performed on *d’* measures, with group as between subjects factor and task and position as within subjects factors. The group effect was not significant, *F*(2, 97) = .12, *p* = .89, and neither were any interactions involving group (*Fs* between .80 and 1.05). There was a significant main effect of task *F*(1, 388) = 13.54, p < .001, η^2^ = .12, with lower *d’* for symbols than letters (mean difference = .11, *p* = < .001). The main effect of position was also significant *F*(4, 388) = 22.89, *p* < .001, η^2^ = .19 and interacted significantly with task *F*(4, 388) = 12.14, *p* < .001, η^2^ = .11, suggesting that the two tasks showed different *d’* as a function of position. Fig 3 presents performance on the five positions for the letter and symbol tasks, separately. We ran the same analysis on the full sample of 177 children, including those who had received non-balanced versions of the VAS tasks. The results were highly similar: The group effect was not significant, *F*(2, 172) = 1.62, *p* = .20, and neither were any interactions involving group (*F*s between .40 and 1.30). There was a significant main effect of task *F*(1, 688) = 21.50, *p* = < .001, η^2^ = .11 and position, *F*(4, 688) = 35.82, *p* < .001, η^2^ = .17. The task x position interaction was also significant, *F*(4, 688) = 11.43, *p* < .001, η^2^ = .06.

Given the negative result on the group effect, we were interested to test whether it was due to a real null result, i.e. evidence in favor of the null hypothesis of equal *d’* of each deficit group vs. TD children, or due to the lack of task sensitivity. To test for the strength of evidence of the null hypothesis, we relied on the Bayes Factor (*B*; [36]), which was calculated in Matlab with the script provided by Z. Dienes (http://www.lifesci.sussex.ac.uk/home/Zoltan_Dienes/inference/Bayes.htm). *Bs* were calculated for the letter and the symbol tasks separately, for each mean difference produced by the comparison of one deficit group vs. TD children. As theory-driven *d’*, the value of .47 was considered as significant group difference [19]. Note that a *B* less than 1/3 represents substantial evidence in favor of the null hypothesis, whereas a *B* higher than 3 is considered in favor of the alternative hypothesis; a *B* between 1/3 and 3 is considered weak or “anecdotal” evidence [36]. As Table 2 shows, all *Bs* were ≤ 1/3, thus in favor of the null hypothesis.

**Table 2:** Mean differences, standard errors and Bayes Factors (B) for the comparison of TD children against each deficit group for the letter and the symbol tasks.

	Letter Task			Symbol Task		
	*Mean Difference*	*Standard Error*	*B*	*Mean Difference*	*Standard Error*	*B*
TD—Dyslexia	.011	.065	.159	.036	.068	.232
TD—SD	.058	.061	.334	-.011	.064	.120

**Fig 4:**
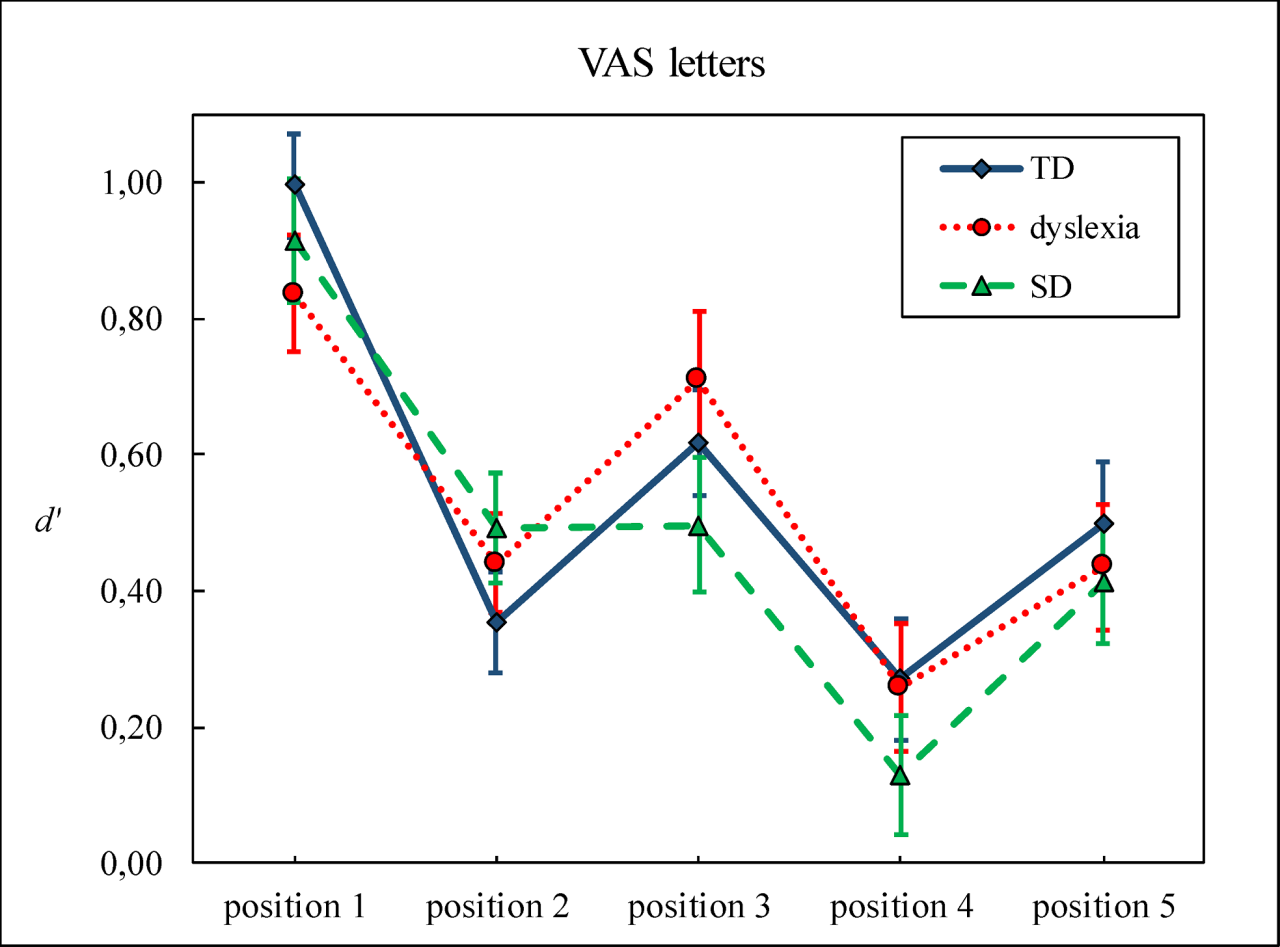
The *d’* of the letter task is shown as a function of position for the three groups. Bars represent standard errors.

**Fig 5:**
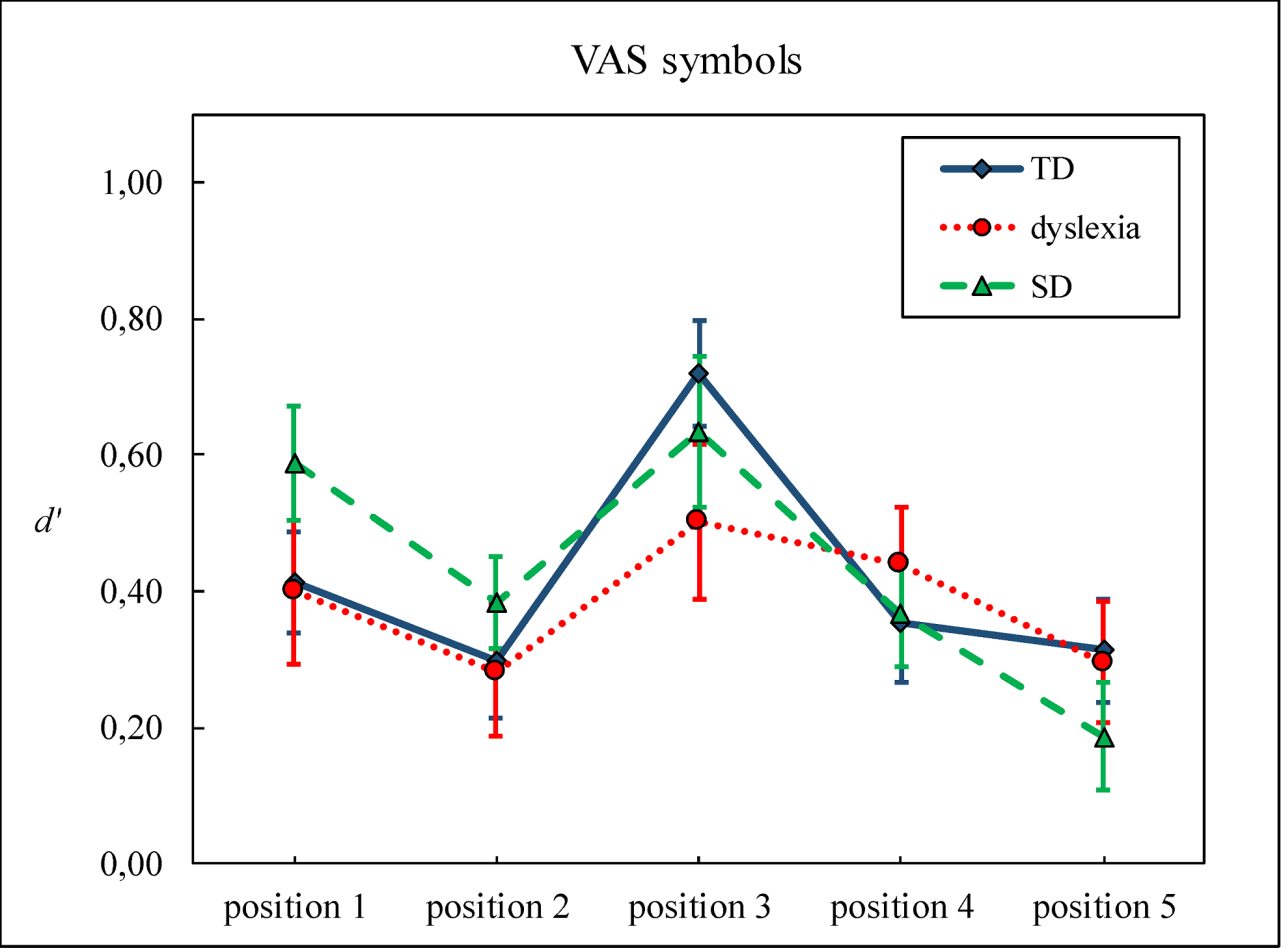
The *d’* of the symbol task is shown as a function of position for the three groups. Bars represent standard errors.

Figs 4 and 5 show *d’* as a function of position for the three groups, for the letter and the symbol tasks, respectively.

To unravel the task x position interaction, two separate ANOVAs for the letter and the symbol tasks were performed, with position as within subjects factor. Both ANOVAs yielded significant effects of position: Letters, *F*(4, 400) = 29.46, *p* < .001, η^2^ = .23; Symbols: *F*(4, 396) = 9.26, *p* < .001, η^2^ = .09. However, pairwise comparisons with Bonferroni correction showed different position-related patterns depending on the task. For letters, position 1 yielded the highest score (*d’* = .93), compared to position 2 (*d’* = .42), position 3 (*d’* = .60), position 4 (*d’* = .22), and position 5 (*d’* = .45, all *ps* < .001). Position 4 had a lower *d’* than position 2, 3, and 5 (all *p*s < .05). For symbols, the highest *d’s* appeared on position 1 (*d’* = .46) and position 3 (*d’* = .64). Position 1 had a higher *d’* than position 5 (*d’* = .27, *p* = .05). Position 3 yielded a higher *d’* than position 2 (*d’* = .32), position 4 (*d’* = .38), and position 5 (all *ps* < .005).

A one-sample t-test against zero showed that even the lowest *d’s* were significantly above chance level, *t*(100) = 4.20, *p* < .001 for position 4 in the letter task ; *t*(99) = 5.71, *p* < .001 for position 5 on the symbol task.

#### Correlates of VAS performance

**Fig 6:**
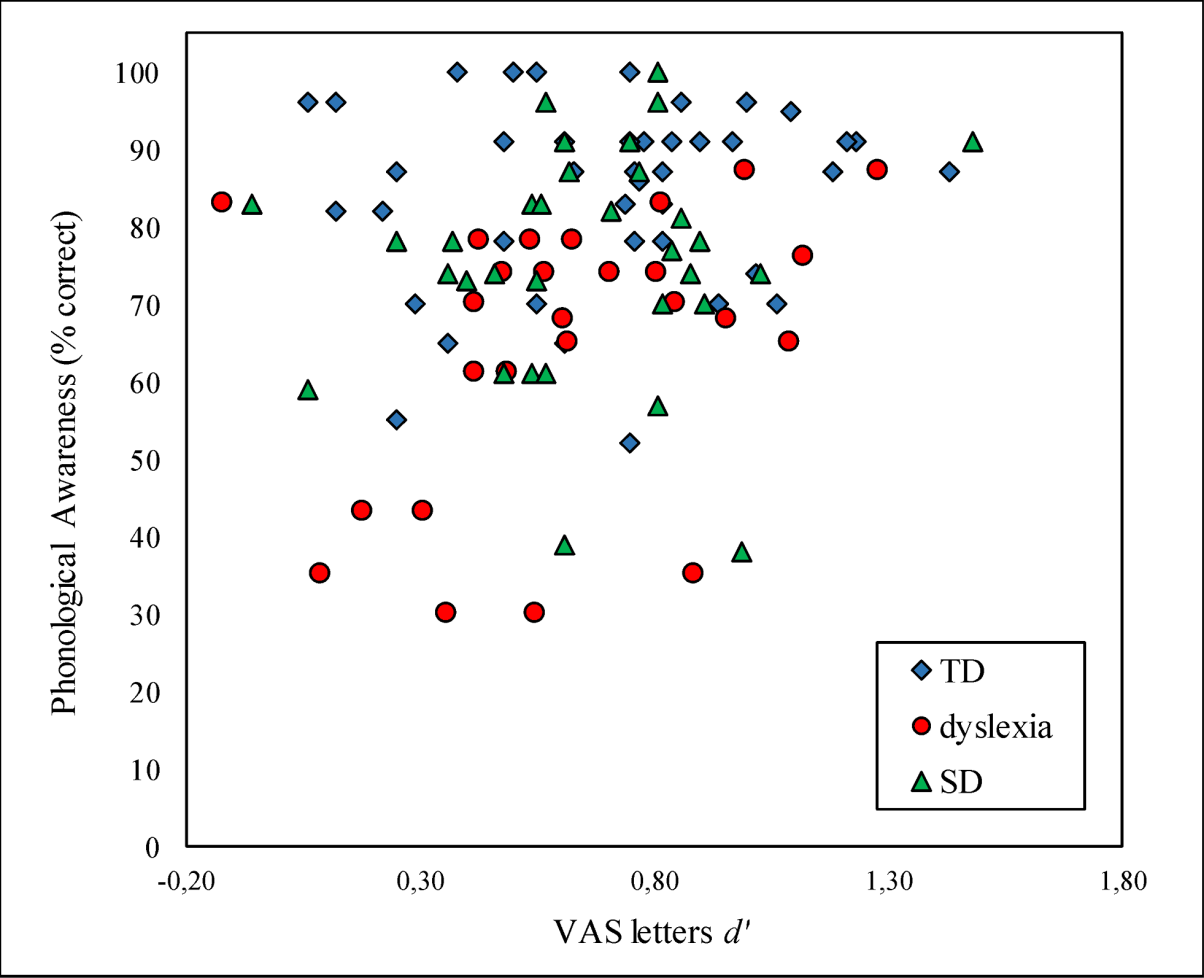
Scatterplot representing the relationship between VAS letters (x axis) and PA (y axis) in the three groups.

The VAS letter and symbol tasks were moderately correlated with each other, r(98) = .43, *p* < .001. Cognitive variables were generally not normally distributed (Kolmogorov-Smirnov test > .09, *p* < .04), with the exception of RAN digits and objects (Kolmogorov-Smirnov test < .08, *p* = .20). The association of VAS performance with cognitive measures was thus tested by means of Spearman correlations. Both VAS tasks showed modest correlations with Digit Span, letters: *r*(99) = .20, *p* = .05, symbols: *r*(98) = .23, *p* = .02, and Symbol Search, letters: *r*(99) = .24, *p* = .02, symbols: *r*(98) = .29, *p* = .003. The symbol task also correlated moderately with RAN objects, *r*(98) = .23, *p* = .02. Given previous reports that VAS and PA contribute independently to reading [5,6], we explored the relation between VAS letters and PA at the level of individual differences in a scatterplot presented in Fig 6. Contrary to expectations, we observed a modest positive correlation, which even showed a trend towards significance, *r*(99) = .18, *p* = .07. Fig 6 shows that children in both deficit groups had highly variable VAS scores. Note, for example, that it was an SD child who showed the highest *d’* on the VAS task. Importantly, it was not the case that children with low PA had high VAS scores and vice versa, as one would expect if their literacy problems are caused by one or the other deficit. The TD group generally showed high PA performance, but also had very heterogeneous VAS scores.

## Discussion

The present study compared VAS in typically developing children with reasonably large and carefully selected samples of children with dyslexia (combined deficits in reading and spelling) and isolated deficits in spelling. We applied rather lenient selection criteria (percentile 20) to identify children with deficits in reading and/or spelling, and we cannot rule out that more severely affected participants would have presented different results. However, most children in the two deficit groups did exhibit serious deficits. On average, children with dyslexia performed below percentile 10 on sentence, word reading and spelling and just above percentile 10 on pseudoword reading. Note that 88% of the children fulfilled the criteria for a dyslexia diagnosis according to the German diagnostic guidelines [35]. Children with SD showed similarly severe impairments in spelling as children with dyslexia, while their reading performance was within the normal range.

The VAS tasks were specifically devised to overcome limitations of global oral reports, thus reducing phonological short-term memory load and possible influences of differences in naming speed. We also added a symbol condition in order to investigate whether the nature of the VAS deficit was verbal rather than visuo-attentional. As a further methodological advancement compared to earlier studies, eye-tracking was applied in order to ensure that children followed the instruction to look at the fixation cross in the middle of the screen at the start of each trial and to rule out the occurrence of eye-movements during presentation. Of note, *d’* was computed on each position for the two tasks, which is a measure of sensitivity representing the distance between the signal (i.e. hit rates) and the signal + noise (i.e. false alarms). We thus consider it a more sensitive measure than simple response accuracy. Based on the calculation of *d’*, we could show that children performed above chance level even in the most difficult condition.

Contrary to the VAS hypothesis of dyslexia [2], the present study yielded no reliable group differences nor any interactions involving group. A Bayes Factor analysis confirmed that the negative result was not due to lack of task sensitivity and that the null hypothesis of absent group differences was the most likely. The absence of a VAS deficit on the symbol task is consistent with previous studies relying on paradigms that did not require oral responses [11,12,16,17,20]. The negative finding in the letter task, however, is not in line with previous evidence, as a number of studies reported lower performance of dyslexic compared to typical readers [11,12]. It is as yet unclear to what extent this reduced VAS performance in participants with dyslexia, and especially in children, might be influenced by inefficient inspection strategies on the five-element string. This confound was excluded in the current study by the applied eye-fixation control, which ensured that the string was fixated on the middle position and induced similarly high performance in all three groups. It is also possible that the verbal “yes/no” response required in our paradigm could inhibit any attempts to name the letters implicitly or explicitly. However, when proper eye-fixation was ensured and covert naming was excluded, no evidence for reduced VAS in dyslexia was found.

Overall, we observed a typical “W”-shaped pattern for letters and “reversed V” shaped pattern for symbols [37]. The typical “W”-shaped pattern in the letter task has been explained by visual crowding particularly affecting positions 2 and 4 [37] and individuals with dyslexia are thought to be more prone to crowding [12]. While the “W”-shaped pattern was replicated in the present experiment, no evidence for excessive crowding was found in the deficit groups, which is consistent with previous evidence [21]

We also replicated the “first letter advantage” as shown in previous forced choice behavioral studies [38–40]. Importantly, this is the first study showing this effect while controlling for eye-fixations, which rules out that participants may have fixated the first instead of the middle position. Such a control is particularly important for children, who may not always follow the instructions to fixate in the middle. The nature of the first-letter-advantage is hotly debated. One position claims that such leftward bias could reflect a change in visual receptive fields for alphabetic stimuli [38, 39]. An alternative explanation calls visual-attention processes into play [40]. While the present study cannot clarify the causal mechanisms underlying this effect, it shows that it is present in both good and poor readers and spellers already after about three years of reading instruction. This finding is consistent with previous cross-sectional evidence on typical developing children in Grades 1 to 6 [41]

For the SD group, the negative result on the VAS task was particularly unexpected. A reduced visual attentional window seemed to be a promising explanation for their obvious deficits in storing word-specific orthographic representations. However, our findings indicate a deficit in phonological awareness but intact VAS in both deficit groups. It has been claimed that VAS and PA make independent contributions to reading [5,6] and that a VAS deficit might represent an alternative explanation for those cases in which a reading impairment is not accompanied by a PA deficit [5]. It follows that if children with dyslexia already show a deficit in PA, as in the present study, they are less likely to manifest also a VAS impairment. The scatterplot in Fig 6, however, does not depict a clear dissociation between PA and VAS skills. The performance on the latter task was rather variable in all three groups. Importantly, there were children in the deficit groups, who showed neither a VAS nor a PA deficit, as well as children with very low PA and VAS scores.

Both letters and symbols tasks showed modest correlations with Digit Span and Symbol Search, indicating an influence of short-term memory and processing speed on task performance. For the children with dyslexia, characterized by an extremely slow and dysfluent reading style, we replicated the often reported association with deficits in RAN [42,43]. It has been argued that RAN and VAS letters might share variance as both might rely on visual-verbal access [11,12,44]. However, our data do not provide evidence in favor of this hypothesis as the VAS letter task did not correlate with RAN. Only the symbol task showed moderate associations with RAN objects (but not letters). Unfortunately, our task battery did not include a measure of visual short-term memory, which might have revealed a stronger contribution to VAS performance than the measure of verbal short-term memory (Digit Span) used in the current study.

In summary, the present study could not replicate previous findings of impaired VAS in dyslexia, and could therefore not confirm any involvement of VAS in reading and spelling. Even the VAS-PA dissociation was not straightforward in our samples of children with dyslexia and isolated spelling impairment. We thus conclude that once strong methodological controls are introduced on tasks, VAS deficits cannot account for reading and spelling deficits in the majority of cases.

## Supporting information

S1 Appendix Studies relying on oral report paradigms are listed in chronological order. (DOCX)

S2 Appendix Studies without oral report paradigms are listed in chronological order. (DOCX)
