# Supplementary material for: Visual attention span performance in German-speaking children with differential reading and spelling profiles: No evidence of group differences
Source: PLoS One. 2018 Jun 18;13(6):e0198903. doi: 10.1371/journal.pone.0198903 (PMC6005485; doi:10.1371/journal.pone.0198903)
Supplement: S1 Appendix — (DOCX) [file pone.0198903.s001.docx]

**S1 Appendix. Studies relying on oral report paradigms are listed in chronological order.**

| **Study** | **Participants** | **Age (years)** | **Language** | **Stimuli** | **Task** | **Results of the group comparison** |
| --- | --- | --- | --- | --- | --- | --- |
| Valdois et al. (2003) [1] | 2 dyslexia | 13-14 | French | five-letter array | Oral whole-report  Oral partial-report | Laurent: good visual processing performance and poor PA  Nicolas: good PA, poor visual processing |
| Hawelka and Wimmer, (2005) [2] | 15 dyslexia  15 TD | 15 | German | two-, four-, six-digit array | Oral partial-report | Group main effect: higher presentation times in dyslexia on the 4- and 6- digits arrays. |
| Hawelka, Huber, and Wimmer (2006) [3] | 12 dyslexia  14 TD | 15  25 | German | five-letter arrays with consonants or digits | Oral partial-report | Group main effect: higher presentation times in dyslexia for consonants and digits. |
| Bosse, Tainturier, and Valdois (2007) [4] | 68 dyslexia  55 TD | 11 | French | five-letter array | Oral whole-report  Oral partial-report | Group main effect: lower accuracy in dyslexia |
|  | 29 dyslexia  23 TD | 10 | English | five-letter array | Oral whole-report  Oral partial-report | Group main effect: lower accuracy in dyslexia |
| Lassus-Sangosse, N’guyen-Morel, and Valdois (2008) [5] | 13 phonological dyslexia  13 non-phonological dyslexia  13 TD | 10 | French | five-letter array | Whole-report after simultaneous or sequential presentation | Group main effect: lower accuracy in dyslexia.  Group x task interaction: in the simultaneous task only, accuracy in non-phonological dyslexia lower than in phonological dyslexia and TD |
| Lobier, Zoubrinetzky, and Valdois (2012) [6] | 14 dyslexia  14 TD | 10 | French | five-letter array | Oral whole-report task | Group main effect: lower accuracy in dyslexia |
| Valdois, Lassus-Sangosse, and Lobier (2012) [7] | 22 dyslexia  22 TD | 10 | French | five-element array of letters, digits or color patches | Oral whole-report | Group main effect: lower accuracy in dyslexia  Group x stimulus interaction: lower accuracy in dyslexia for letters and digits only. |
|  | 24 dyslexia  24 TD | 10 | French | five-letter array | Oral whole-report with/without concurrent articulation | Group main effect: lower accuracy in dyslexia.  Task main effect: lower accuracy for the concurrent articulation task, but no group x task interaction |
| Germano, Reilhac, Capellini, and Valdois (2014) [8] | 33 dyslexia  33 TD | 10 | Portuguese | five-letter string | Oral whole-report | Group main effect: lower accuracy in dyslexia |
| Zoubrinetzky, Bielle, and Valdois (2014) [9] | 14 dyslexia-VAS deficit  14 dyslexia-PA deficit  14 TD-CA  14 TD-RL | 10 | French | five-letter string | Oral whole-report  Oral partial-report | Group main effect: lower accuracy in the dyslexia-VAS deficit group compared to both TD groups |
| Lallier, Valdois, Lassus-Sangosse, Prado, and Kandel(2014) [10] | 9 dyslexia  9 TD | 11 | bilingual French-Spanish | five-letter string | Oral whole-report  Oral partial-report | Group main effect: lower accuracy in dyslexia |
| Lallier, Thierry, Barr, Carreiras, and Tainturier(2018) [11] | 15 dyslexia  15 TD | 20 | monolingual English | five-letter string | Oral partial-report | No significant group main effect |
|  | 15 dyslexia  15 TD | 24 | Welsh-English  bilinguals |  |  |  |
| Zoubrinetzky, Collet, Serniclaes, Nguyen-Morel, and Valdois (2016) [12] | 63 dyslexia  63 TD | 10 | French | five-letter string | Oral whole-report  Oral partial-report | *F* and *p* statistics for the comparison with TD not reported.  cit. “The dyslexic group’s VA span abilities were slightly lower than for the TD children (<-1 SD) but here again some children showed a clear VA span disorder while others performed within the normal range”, p. 9 |
| Yeari, Isser, and Schiff (2017) [13]  Experiment 1 | 24 dyslexia  26 TD | 28  26 | Hebrew | five-letter string | Oral whole-report task | Group main effect: lower accuracy in dyslexia |

*Note*. TD: typically developing children; TD-CA: typically developing children matched on chronological age; TD-RL: typically developing children matched on reading level.

**References**

[1] Valdois S, Bosse M-L, Ans B, Carbonnel S, Zorman M, David D, et al. Phonological and visual processing deficits can dissociate in developmental dyslexia: Evidence from two case studies. Read Writ. 2003;16: 541–572. doi: 10.1023/A:1025501406971

[2] Hawelka S, Wimmer H. Impaired visual processing of multi-element arrays is associated with increased number of eye movements in dyslexic reading. Vision Res. 2005;45(7): 855–63. doi: 10.1016/j.visres.2004.10.007

[3] Hawelka S, Huber C, Wimmer H. Impaired visual processing of letter and digit strings in adult dyslexic readers. Vision Res. 2006;46(5):718–23. doi: 10.1016/j.visres.2005.09.017

[4] Bosse ML, Tainturier MJ, Valdois S. Developmental dyslexia: The visual attention span deficit hypothesis. Cognition. 2007;104(2): 198–230. doi: 10.1016/j.cognition.2006.05.009

[5] Lassus-Sangosse D, N’guyen-Morel MA, Valdois S. Sequential or simultaneous visual processing deficit in developmental dyslexia? Vision Res. 2008;48(8): 979–88. doi: 10.1016/j.visres.2008.01.025

[6] Lobier M, Zoubrinetzky R, Valdois S. The visual attention span deficit in dyslexia is visual and not verbal. Cortex. 2012;48(6): 768–773. doi: 10.1016/j.cortex.2011.09.003

[7] Valdois S, Lassus-Sangosse D, Lobier M. Impaired letter-string processing in developmental dyslexia: What visual-to-phonology code mapping disorder? Dyslexia. 2012;18(2): 77–93. doi: 10.1002/dys.1437

[8] Germano GD, Reilhac C, Capellini SA, Valdois S. The phonological and visual basis of developmental dyslexia in Brazilian Portuguese reading children. Front Psychol. 2014;5: 1169. doi : 10.3389/fpsyg.2014.01169

[9] Zoubrinetzky R, Bielle F, Valdois S. New insights on developmental dyslexia subtypes : Heterogeneity of mixed reading profiles. PLoS One. 2014;9(6): e99337. doi: 10.1371/journal.pone.0099337

[10] Lallier M, Valdois S, Lassus-Sangosse D, Prado C, Kandel S. Impact of orthographic transparency on typical and atypical reading development : Evidence in French-Spanish bilingual children. Res Dev Disabil. 2014; 35: 1177-1190. doi: 10.1016/j.ridd.2014.01.021

[11] Lallier M, Thierry G, Barr P, Carreiras M, Tainturier M. Learning to read bilingually modulates the manifestation of dyslexia in adults. Sci Stud Read. 2018;22(4): 335-349. doi: 10.1080/10888438.2018.1447942

[12] Zoubrinetzky R, Collet G, Serniclaes W, Nguyen-Morel MA, Valdois S. Relationships between categorical perception of phonemes, phoneme awareness, and visual attention span in developmental dyslexia. PLoS One. 2016;11(3): 1–26. doi: 10.1371/journal.pone.0151015

[13] Yeari M, Isser M, Schiff R. Do dyslexic individuals present a reduced visual attention span ? Evidence from visual recognition tasks of non-verbal multi-character arrays. Ann Dyslexia. 2017;67: 128–146. doi: 10.1007/s11881-016-0132-4
