# Supplementary material for: Visual attention span performance in German-speaking children with differential reading and spelling profiles: No evidence of group differences
Source: PLoS One. 2018 Jun 18;13(6):e0198903. doi: 10.1371/journal.pone.0198903 (PMC6005485; doi:10.1371/journal.pone.0198903)
Supplement: S2 Appendix — (DOCX) [file pone.0198903.s002.docx]

**S2 Appendix. Studies without oral report paradigms are listed in chronological order**

| **Study** | **Participants** | **Age (years)** | **Language** | **Stimuli** | **Task** | **Results on the group comparison** |
| --- | --- | --- | --- | --- | --- | --- |
| Pammer, Lavis, Hansen, and Cornelissen(2004) [1] | 13 dyslexia  13 TD | 10 | English | five-symbol array | 2AFC between two arrays | Group main effect: lower *d’* in dyslexia |
| Shovman and Ahissar (2006) [2] | 20 dyslexia  20 TD | 23  24 | Hebrew | Letters from the Georgian alphabet  Single/triple-element string, large/small, high/low contrast uniform/noisy background brief/long SOA | 4AFC | No significant group main effect nor group x condition interaction |
| Hawelka and Wimmer (2008) [3] | 18 dyslexia  18 TD | adults | German | five-element arrays of letters or pseudo-letters with high/low similarity | Probe task with spacebar-press in case the string contained the probe | No group main effect on error rates and detection times |
| Jones, Branigan, and Kelly(2008) [4] | 19 dyslexia  19 TD | 22 | English | five-symbol array | 2AFC between two arrays | Group main effect: lower accuracy in dyslexia |
| Ziegler, Pech-Georgel, Dufau, and Grainger(2010) [5] | 28 dyslexia  29 TD | 10 | French | five-element array of letters, digits or symbols | Partial-report with 2AFC on a cued position | Group x stimulus interaction: lower accuracy in dyslexia for letters and digits, but not for symbols. |
| Collis, Kohnen, and Kinoshita(2013) [6] | 18 dyslexia  23 TD | 22 | English | 5-element string of letters, digits, symbols | Partial-report with 9AFC | Trend for a Group main effect on % errors.  Stimuli x Group interaction: dyslexia worse than TD on digits and letters but not on symbols. |
| Yeari, Isser, and Schiff (2017) [7]  Experiment 1 | 24 dyslexia  26 TD | 28  26 | Hebrew | seven open and seven closed symbols | AR task  CR task  High/low discriminability | No significant group main effect nor group x conditions interaction |
| Yeari, Isser, and Schiff (2017) [7]  Experiment 2 | 16 dyslexia  19 TD | 28  25 | Hebrew | Straight-line characters in AR task;  Nine unfamiliar symbols in CR | AR task  CR task  Presentation time: 100 ms or 200 ms | No significant group main effect nor group x conditions interaction |

*Note*. TD: typically developing children; AFC: alternative forced choice task; AR task: array recognition task; CR task: character recognition task.

**References**

1. Pammer K, Lavis R, Hansen P, Cornelissen PL. Symbol-string sensitivity and children’s reading. Brain Lang. 2004;89(3): 601–610. doi: 10.1016/j.bandl.2004.01.009
2. Shovman MM, Ahissar M. Isolating the impact of visual perception on dyslexics’ reading ability. Vision Res. 2006;46(20): 3514–3525. doi: 10.1016/j.visres.2006.05.011
3. Hawelka S, Wimmer H. Visual target detection is not impaired in dyslexic readers. Vision Res. 2008;48(6): 850–852. doi: 10.1016/j.visres.2007.11.003
4. Jones MW, Branigan HP, Kelly ML. Visual deficits in developmental dyslexia: Relationships between non-linguistic visual tasks and their contribution to components of reading. Dyslexia. 2008;14: 95–115. doi: 10.1002/dys
5. Ziegler JC, Pech-Georgel C, Dufau S, Grainger J. Rapid processing of letters, digits and symbols: What purely visual-attentional deficit in developmental dyslexia? Dev Sci. 2010;13(4): 8–14. doi: 10.1111/j.1467-7687.2010.00983.x
6. Collis NL, Kohnen S, Kinoshita S. The role of visual spatial attention in adult developmental dyslexia. Q J Exp Psychol. 2013;66(2): 245–260. doi: 10.1080/17470218.2012.705305
7. Yeari M, Isser M, Schiff R. Do dyslexic individuals present a reduced visual attention span? Evidence from visual recognition tasks of non-verbal multi-character arrays. Ann Dyslexia. 2017;67: 128–146. doi: 10.1007/s11881-016-0132-4
